# Targeting retinal dopaminergic neurons in tyrosine hydroxylase-driven green fluorescent protein transgenic zebrafish

**Published:** 2008-12-26

**Authors:** Shi Meng, Soojin Ryu, Bin Zhao, Dao-Qi Zhang, Wolfgang Driever, Douglas G. McMahon

**Affiliations:** 1 Department of Biological Sciences, Vanderbilt University, Nashville, TN; 2 Developmental Biology, Institute of Biology 1, Faculty of Biology, University of Freiburg, Freiburg, Germany

## Abstract

**Purpose:**

Dopamine plays key roles in a variety of basic functions in the central nervous system. To study developmental and functional roles of dopaminergic cells in zebrafish, we have generated a transgenic line of zebrafish expressing green fluorescent protein (GFP) under the control of the tyrosine hydroxylase (*th1*) promoter.

**Methods:**

A 12 kb gene fragment that contains the *th1* promoter was isolated and ligated to the MmGFP coding sequence, linearized, microinjected into 1–2 cell stage embryos and the founders crossed with wild-type fish to screen for transgenic lines. *Tg(−12th:MmGFP)* embryos were visualized under fluorescence microscopy for GFP expression during development. Confocal microscopy was used to visualize GFP-labeled cells in the living whole mount retina and immunostained vertical sections of adult zebrafish retina. Single-cell reverse transcription polymerase chain reaction (RT–PCR) was performed on individual GFP+ cells collected from dispersed retinal cell cultures for *th1* and dopamine transporter (*dat*). Loose-patch recordings of spike activity of GFP+ neurons were made in isolated whole mount retinas.

**Results:**

*th1* promoter-driven GFP exhibited robust expression in the brain and retina during zebrafish development. In juvenile and adult fish retinas, GFP was expressed in cells located in the inner nuclear layer. Immunocytochemistry with antibodies for GFP and TH showed that 29±2% of GFP-labeled cells also expressed TH. Two subpopulations of GFP-labeled cells were identified by fluorescent microscopy: bright GFP-expressing cells and dim GFP-expressing cells. Seminested single-cell RT–PCR showed that 71% of dim GFP-expressing cells expressed both *th* and *dat* mRNA. Loose-patch voltage-clamp recording from dim GFP-labeled cells in retinal whole mounts revealed that many of these dopaminergic neurons are spontaneously active in darkness.

**Conclusions:**

Although this *Tg(−12th:MmGFP)* line is not a completely specific reporter for dopaminergic neurons, using relative GFP intensity we are able to enrich for the selection of retinal dopaminergic cells in vitro and in situ in molecular and electrophysiological experiments. This transgenic line provides a useful tool for studying retinal dopaminergic cells in the zebrafish.

## Introduction

In the central nervous system, dopamine (DA) plays important roles in modulating a variety of physiologic events such as movement, emotion, memory, and reward processing. In the vertebrate retina, dopamine is involved in mediating neuronal adaptation to light [1,2], and circadian rhythmicity [3-5], as well as cell survival and eye growth [6,7]. In teleost retinas, dopamine is released by dopaminergic interplexiform cells (DA-IPCs), which contact horizontal and bipolar cell dendrites in the outer plexiform layer (OPL), and receive input from amacrine and bipolar cell terminals in the inner retina [1,8-10]. DA-IPCs have been proposed to be a centrifugal pathway for information flow from the inner to the outer retina [9,11], and they have been shown to mediate the modulatory effect of olfactory input on retinal ganglion cell activity [12].

Despite the diverse roles of DA cells in retinal functions, the understanding of DA cell function has been limited because they have a low density in the retina and cannot be identified in living retina by morphological characteristics [13,14]. In the mouse, transgenic lines have been created, in which reporter genes are driven by the promoter for the tyrosine hydroxylase (*th*) gene, the rate-limiting enzyme for dopamine biosynthesis. These transgenic lines provide strategies to identify dopaminergic neurons in vitro [15] and in situ in living retina [16]. Here we report marking dopaminergic neurons in vivo in zebrafish retina using a similar approach.

Zebrafish (*Danio rerio*) has become of interest for research on neurogenesis and the dopaminergic system due to fast embryonic development, as well as the availability of mutagenesis and transgenesis. For example, tyrosine hydroxylase immunoreactivity studies have revealed the presence of dopaminergic neurons in the ventral diencephalon as early as 18 h post fertilization (hpf) [17], and in the retina at three days post fertilization (dpf) [18]. In addition, mutant zebrafish lines in which catecholaminergic or dopaminergic system development is disrupted have been isolated using large-scale mutagenesis screens [19-23]. Studies of these mutants can enhance our understanding of the dopaminergic system development and function. To investigate the normal function and morphology of living DA cells in zebrafish retinas, we established a transgenic zebrafish model in which GFP is driven by sequences of the zebrafish *th1* promoter. Here, we report morphological, molecular, and physiologic characterization of the genetically labeled neurons in this transgenic zebrafish line.

## Methods

### Transgenic zebrafish

To generate the *Tg(−12th:MmGFP)* transgenic zebrafish, we isolated a genomic P1-derived artificial chromosome (PAC) clone, BUSMP706E03252Q3, containing the zebrafish tyrosine hydroxylase 1 (*th1*) gene promoter region by screening the zebrafish PAC library (BUSMP706) with a probe containing a part of *th1* cDNA sequence. To identify genomic fragments containing the *th1* promoter region, we digested the PAC clone with several restriction enzymes and performed duplicate Southern hybridization using two probes. The first probe was a genomic PCR product of the 5′UTR region of the *th1* locus. Following identification of the *th1* 5′UTR by RACE, we designed two primers as shown in Table 1 for the genomic PCR. The second probe was generated from a previously published partial *th1* cDNA clone [17] by PvuII-BglII digest and contained approximately 100 bp of carboxyterminal portion of the coding region of the *th1* gene. To identify a genomic fragment, which contains mostly the promoter region and not the coding region, we sought to isolate PAC restriction fragments, based on Southern analysis, that are positive for the 5′UTR probe but are negative for the carboxyterminal probe. A XbaI-XhoI fragment was identified, which fulfilled this criterion. The XbaI-XhoI fragment was further digested with EcoRI (EcoRI restriction site is found immediately downstream of the Th1 start ATG) and XhoI, and was cloned into a pBluescript II vector. The end sequencing of this fragment using T7 primer revealed that the fragment contains the *th1* genomic region of chromosome 25 starting at position 20376290 (Ensembl Zv7 assembly). Since the *th1* transcript begins at the position 20364304, and since *th1* is oriented in reverse direction on this chromosome, the EcoRI-XhoI fragment encompasses 11986 bp of *th1* genomic promoter region; therefore, we refer to this fragment as *th1* 12 kb promoter fragment. The MmGFP chromophore coding region and SV40 polyA sequence was PCR-amplified from pG1 vector (gift of Chi-Bin Chien and Darren Gilmour, University of Utah, Salt Lake City, UT and EMBL) and cloned downstream of the *th1* 12 kb promoter fragment in the pBluescript II vector. To increase the translational efficiency, we adjusted the translational initiation context to better match the consensus Kozak sequence (GCCATGG). Transgenic zebrafish were generated by microinjection of 1–2 nl of 50 ng/μl linearized DNA into 1- to 2-cell stage embryos (University of Freiburg, Freiburg, Germany). The founders were crossed with wild-type fish and their progeny were screened to establish transgenic lines. The transgene integration used for this study has the allele designation *Tg(−12th:MmGFP)^m899^.*

**Table 1:** Primers for the genomic PCR and for the nested PCR

**Primer**	**Sequence**
TH5RaceM13FF	5′-GTTTACATTTCCAATGCGCGCG-3′
TH5RaceM13FR	5′-ATATCAGCAAAAGAGATTTAATGAAG-3′
	
*th* forward primer	5′-CAGGATTTCAGTTGAGGCCAGTG-3′
*th* reverse primer	5′-ATGCACCAGCTCTCCATAGGATG-3′
*th* nested primer	5′-TCCACAGTGAACCAGTACATTGTCG-3′
	
*dat* forward primer	5′-AACTCTCTGACCAGCTTCTCCTCCG-3′
*dat* reverse primer	5′-ATTCCACCGTTGGTGACGCAG-3′
*dat* nested primer	5′-GTCGATGAGTCCCGTGATCAC-3′

### Screen of transgenic offspring

*Tg(−12th:MmGFP)* transgenic zebrafish hemizygous for the *Tg(−12th:MmGFP)* transgene were crossed with wild-type AB* fish to produce the hemizygous *Tg(−12th:MmGFP)* fish used in this study. Eggs were collected in the morning, 1 h after mating, and treated with 0.003% (w/v) phenyl-2-thiourea (PTU; Sigma, St. Louis, MO) at 24 hpf to prevent the development of melanin pigment. The transgenic offspring were screened by visualizing GFP expression at 24–30 hpf with a fluorescent microscope (Leica MZFLIII, Leica Microsystems, Bannockburn, IL). The fish were treated in accordance with National Institutes of Health and Vanderbilt University Division of Animal Care guidelines.

### Imaging

*Tg(−12th:MmGFP)* expression in the zebrafish embryos was visualized using a fluorescent dissecting microscope (Leica MZFLIII) connected to a QImaging Retiga 1300 CCD digital camera. The bright-field or fluorescent images were captured using QCapture v 1.2.0 software. All other images, including whole mount retina and immunostained vertical sections of adult zebrafish retina, were visualized with a laser scanning confocal microscope (Zeiss LSM5 Pascal, Carl Zeiss, Jena, Germany) at excitation wavelengths of 488 nm or 543 nm. The z-stack images were scanned at an optical slice of 4 μm for whole-retina expression in embryos 3 dpf and 1 μm for individual cells in living retina. For cell counting on complete retinas, tile-scans of 16 images, measuring 512×512 µm^2^, were collected from double immunostained whole mount retinas.

### Immunocytochemistry

Transgenic fish (approximately 1–3 months old) were kept in darkness overnight and then euthanized on ice. Both eyes were removed from the fish and then hemisected to remove the cornea and lens. The eyecups were fixed for 2 h and then rinsed three times for 5 min each in phosphate buffered saline (PBS; 137 mM NaCl, 2.6 mM KCl, 5.4 mM Na_2_HPO_4_, 1.8 mM KH_2_PO_4_), pH 7.4, cryoprotected in 30% sucrose overnight, and cryosectioned at 15 µm. Cryostat sections were then blocked with 1% bovine serum albumin (BSA) and 0.3% Triton X-100 in 0.1 M PBS for 2 h, and stained with 1:20,000 rabbit anti-GFP (Molecular Probes, Carlsbad, CA), and 1:2,000 sheep anti-TH (Chemicon, Temecula, CA) primary antibodies overnight at room temperature. After rinsing, a secondary incubation was performed for 2 h in a mixture containing 1:500 cy3-conjugated donkey antirabbit IgG(H^+^L) and 1:500 cy5-conjugated donkey antisheep IgG(H^+^L) antibodies (Molecular Probes). Whole mount retina immunocytochemistry was performed similarly, except that isolated retinas, instead of whole eyecups, were processed for staining, and the retinas were incubated in primary antibodies for 48 h.

### Single-cell RT–PCR

To make dispersed cell cultures, we isolated retinas in Leibovitz’s L-15 medium (Gibco, Invitrogen, Carlsbad, CA). These were digested with 20 units/ml papain for 20 min and gently triturated in L-15 medium. The cell suspensions were distributed into 35 mm culture dishes, which were kept at room temperature for 30 min. For collection, cells were observed with an inverted microscope (Nikon, Lewisville, TX), and perfusion system was used to avoid contamination [24]. The inlet tube perfused the target cell with an extracellular solution for zebrafish that contained 137 mM NaCl, 2.5 mM KCl, 2.5 mM MgCl_2_, 2.5 mM CaCl_2_, 10 mM HEPES, and 10 mM glucose (pH 7.4). Individual cells were aspirated into the pipette and after washing with extracellular solution, the tip of the pipette harboring the target cell was broken into a 0.5 ml tube with 14 μl of Master Mix 1 and maintained on dry ice. Reverse transcription (RT) was performed using SuperScript II Reverse Transcriptase (Invitrogen, Carlsbad, CA) according to manufacturer’s recommendations. For seminested polymerase chain reaction (PCR), we used the primers shown in Table 1. Seminested PCR was performed using an Eppendorf Mastercycler gradient thermocycler (Eppendorf, Hamburg, Germany). The first round reaction was incubated at 94 °C for 5 min, then at 94 °C for 30 s, 50 °C for 30 s, and 72 °C for 30 s for 15 cycles, followed by 72 °C for 10 min. Nested-PCR reactions were performed using 1 μl of the first round PCR product in a 100 μl sample. The second round reaction was performed at 94 °C for 5 min, then at 94 °C for 30 s, 55 °C for 30 s, and 72 °C for 30 s for 30 cycles, followed by 72 °C for 10 min.

### Electrophysiological recordings

*Tg(−12th:MmGFP)* zebrafish were dark-adapted overnight before the day of recording. The separation of retina was performed in oxygenated extracellular solution under dim red light. The extracellular solution contained the following: 116 mM NaCl, 2.4 mM KCl, 1.2 mM CaCl_2_, 1.2 mM MgCl_2_, 1 mM NaH_2_PO_4_, 28 mM NaHCO_3_, 10 mM glucose. This solution was bubbled with a gaseous mixture of 95% O_2_ and 5% CO_2_. The retina was placed photoreceptor-side down in the recording chamber and maintained in the dark for at least 1 h before recording. The chamber was then mounted on the stage of an upright conventional fluorescent microscope (Leica DM LFSA; Leica Microsystems, Wetzlar, Germany). Oxygenated medium was continuously perfused into the recording chamber at a rate of roughly 2–3 ml/min, and the recording was performed at approximately 21 °C.

During recording, GFP+ cells were identified by fluorescence microscopy using a brief illumination of fluorescent excitation light. GFP-labeled cells and glass electrodes were visualized using infrared differential interference contrast (DIC) optics for recording. The glass electrodes were prepared using DMZ universal puller (Zeitz-Instrumente, Munich, Germany) and filled with a solution containing 150 mM NaCl and 10 mM HEPES (pH 7.5). The resistance of pipettes filled with this solution was 9–10 MΩ. The recording was made from the soma of GFP-labeled cells using a pipette with a holding potential of 0 mV. Experiments were executed and data were recorded using Clampex 8.0 software connected to an AxoClamp-2B amplifier (Molecular Devices, Palo Alto, CA), via a digitizer 1322A (Molecular Devices). Data were analyzed with Clampfit 9.2 (Molecular Devices). Action potential frequency was calculated from 40 s recordings from each cell. Values are mean ± standard deviation.

## Results

### Generation of *Tg(−12th:MmGFP)* transgenic fish

**Figure 1:**
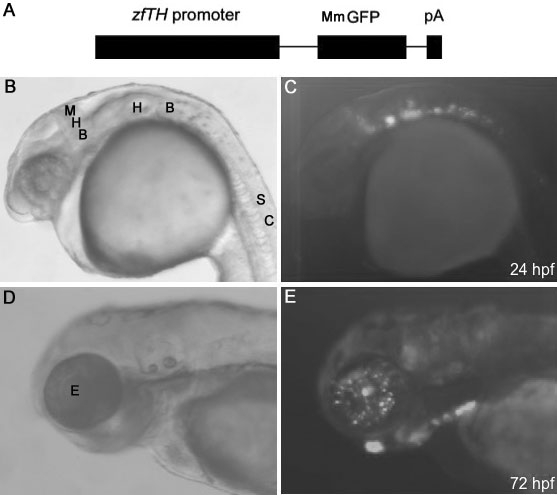
*TH* promoter-driven GFP expression during zebrafish development. **A:** Schematic map of the *Tg(−12th:MmGFP)* construct. The 12 kb sequence containing the zebrafish *TH* (*zfTH)* promoter was ligated with MmGFP followed by an SV40 polyadenylation tail (pA). **B** and **C** display the bright-field image, and fluorescence image of GFP expression in the embryonic neural system at 24 hpf, respectively. **D** and **E** illustrate the bright-field image of the embryonic zebrafish, and fluorescence image of GFP expression in the embryonic retina at 72 hpf, respectively. For **B** – **E**, dorsal is on the top and anterior is on the left. Abbreviations: Midbrain and hindbrain boundary (MHB); hindbrain (HB); spinal cord (SC); eye (E).

An approximately 12 kb genomic fragment containing the zebrafish tyrosine hydroxylase gene *th1* promoter was isolated and ligated with green fluorescence protein (MmGFP) followed by a SV40 polyadenylation tail (pA; Figure 1A). The recombinant DNA was injected into 1–2 cell zebrafish embryos that were allowed to grow to adulthood. The founder fish were then crossed with wild-type fish and screened for germ-line transmission of the transgene. Larval fish with GFP expression were raised as transgenic F1 fish.

### *th*-driven GFP expression during development

**Figure 2:**
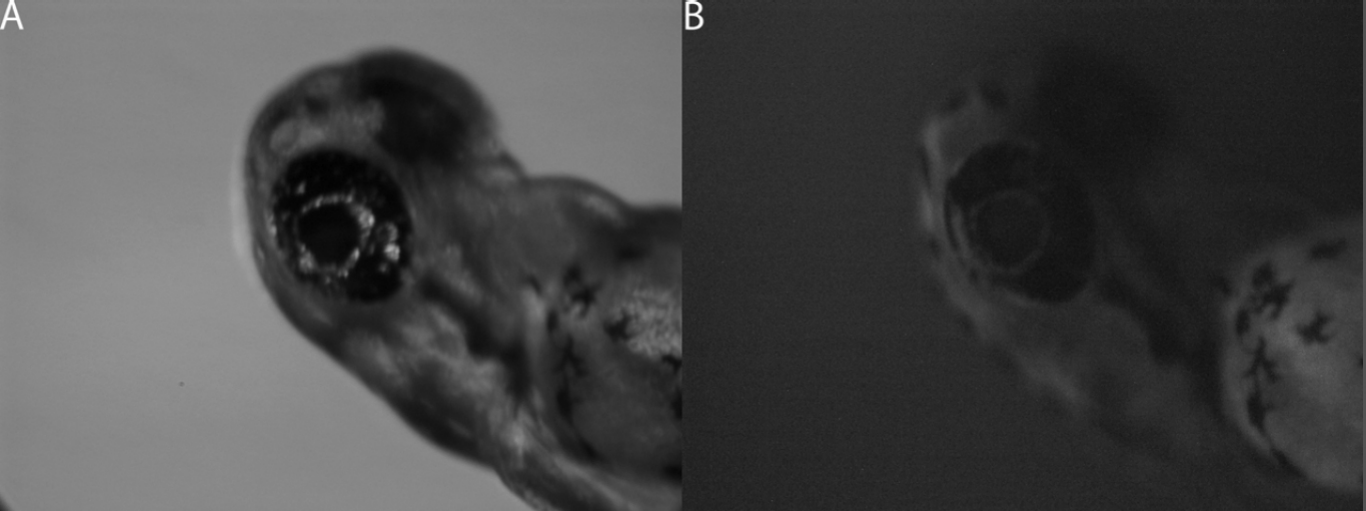
Lack of GFP expression in wild-type zebrafish. Panel **A** illustrates the bright-field image of embryonic zebrafish at 72 hpf. A fluorescence image from the same zebrafish shows no GFP expression in the retina.

To determine the GFP expression pattern in these *Tg(−12th:MmGFP)* fish, we crossed hemizygous transgenic F1 fish with wild-type fish and examined GFP expression in their progeny using fluorescence microscopy. The transgenic embryos showed robust GFP expression in the embryonic brain region, including the midbrain and hindbrain boundary, as well as the hindbrain and spinal cord at 24 hpf (Figure 1C). At 72 hpf, individual neurons exhibiting GFP fluorescence were identified within the retina (Figure 1E). Imaging of nontransgenic wild-type fish at 72 hpf revealed a lack of GFP fluorescence (Figure 2).

### Colocalization of GFP with TH

**Figure 3:**
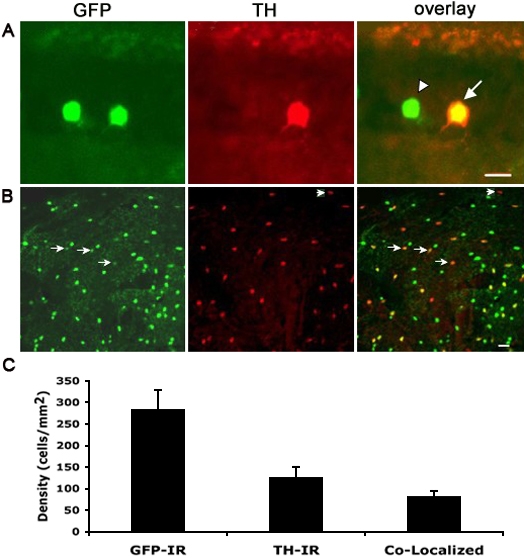
Double immunostaining using anti-GFP and anti-TH antibodies. Immunostaining experiments were performed in vertical retinal sections **(A)** and the whole-mount retina **(B).** GFP-IR, TH-IR, and GFP-IR/TH-IR are shown in green, red and yellow, respectively. Quantification of double-staining in the whole-mount retina is shown in **C.** Values represent the mean±SD from 4 individual retinas. The cell density was calculated by dividing the number of cells by the image area calculated by MetaMorph. Overall, 29±2% of GFP-labeled cells coexpressed TH. Scale bar equals10 μm for **A** and 20 μm for **B**. Arrowhead in **A** points to a GFP-IR/non-TH-IR cell. Arrowhead in **B** points to a TH-IR/non-GFP-IR cell.

**Figure 4:**
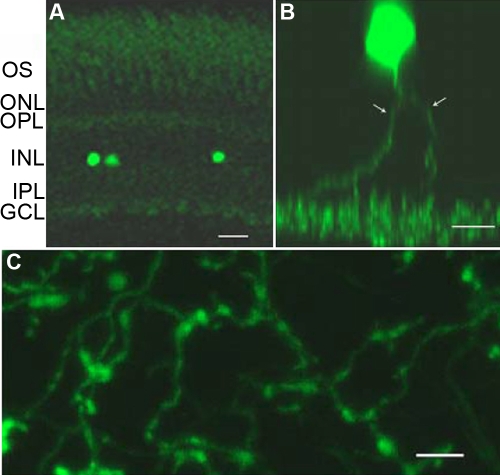
Morphology of GFP-expressing cells in the retina. **A:** Localization of GFP-positive cells can be seen in this vertical retinal section. Most of the GFP-expressing cells were found at the proximal cellular row of the inner nuclear layer. Abbreviations: ONL: outer nuclear layer (ONL); OPL: outer plexiform layer (OPL); INL: inner nuclear layer; IPL: inner plexiform layer. **B:** This z-stack image shows the somata and processes of a single cell. **C,** GFP fluorescent fiber network in the inner plexiform layer. Scale bar equals 20 μm for **A** and **C,** and 10 μm for **B**.

To determine the overlap of *-12th:MmGFP* with native TH expression, we performed double-labeled immunocytochemistry using antibodies for TH and GFP on vertical sections and whole mount adult retinas. Figure 3A shows a vertical section in which GFP was expressed within two cells in the inner nuclear layer (INL). The cell on the right was co-labeled with TH antibody (arrow), while the one on the left was not (arrowhead). GFP+ cells were uniform in their morphological characteristics with cell bodies located in the proximal cellular row of the inner nuclear layer and branched processes extending to an extensive fiber network in the inner plexiform layer (Figure 4). Application of GFP antibody to nontransgenic wild-type zebrafish, or application of secondary antibodies alone without primary TH or GFP antibodies, resulted in a lack of staining. Immunocytochemistry of whole mount retinas was performed to examine the colocalization of GFP-immunoreactivity (IR) and TH-IR (Figure 3B). Arrows indicate cells that express both GFP and TH. Note that while the majority of TH-IR cells also expressed GFP, there were cells single-labeled by TH antibody (arrowhead). To quantify the colocalization rate of TH and GFP in the whole retina, we processed four whole-mount retinas from 4 fish for immunocytochemistry, and we analyzed the images of each entire retina. On average, 29.2 ± 1.7% (mean ± SD, n=4) of GFP-expressing neurons also expressed TH, and GFP-expressing neurons exhibited a density of 286 ± 43 cells/mm^2^. Meanwhile, TH-expressing cells had a density of 129 ± 21 cells/mm^2^ throughout the retina and 65.0 ± 3.4% of TH-expressing cells were labeled by GFP. Images of entire retinas that had been double-labeled for GRP-IR and TH-IR showed that both GFP-IR neurons and TH-IR neurons had a relatively even distribution throughout the retinas (data not shown).

**Figure 5:**
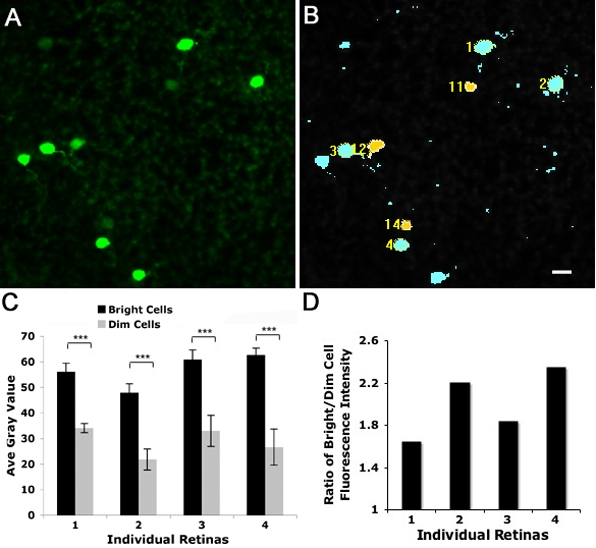
Quantification of fluorescence intensity among GFP-labeled cells. **A:** Representative GFP-immunostained cells shown in **A** were analyzed in MetaMorph (**B**). The scale bar represents 10 μm for **A** and **B**. Bright cells were color coded as blue (cells 1, 2, 3 and 4), and dim cells were yellow (cells 11, 12, and 14). **C:** Quantification of fluorescence intensity of bright and dim cells were analyzed in four individual retinas. For each retina, 10 bright cells and 10 dim cells were chosen using the same threshold. Value represents mean±SD (n=10 for each cell subpopulation). Triple asterisks (***) indicate p<0.001. **D** displays the ratio of bright cell fluorescence to dim cell fluorescence intensity. For all four retinas, bright cells were 2.0±0.3 (mean±SD) fold brighter than dim cells in fluorescence intensity.

**Figure 6:**
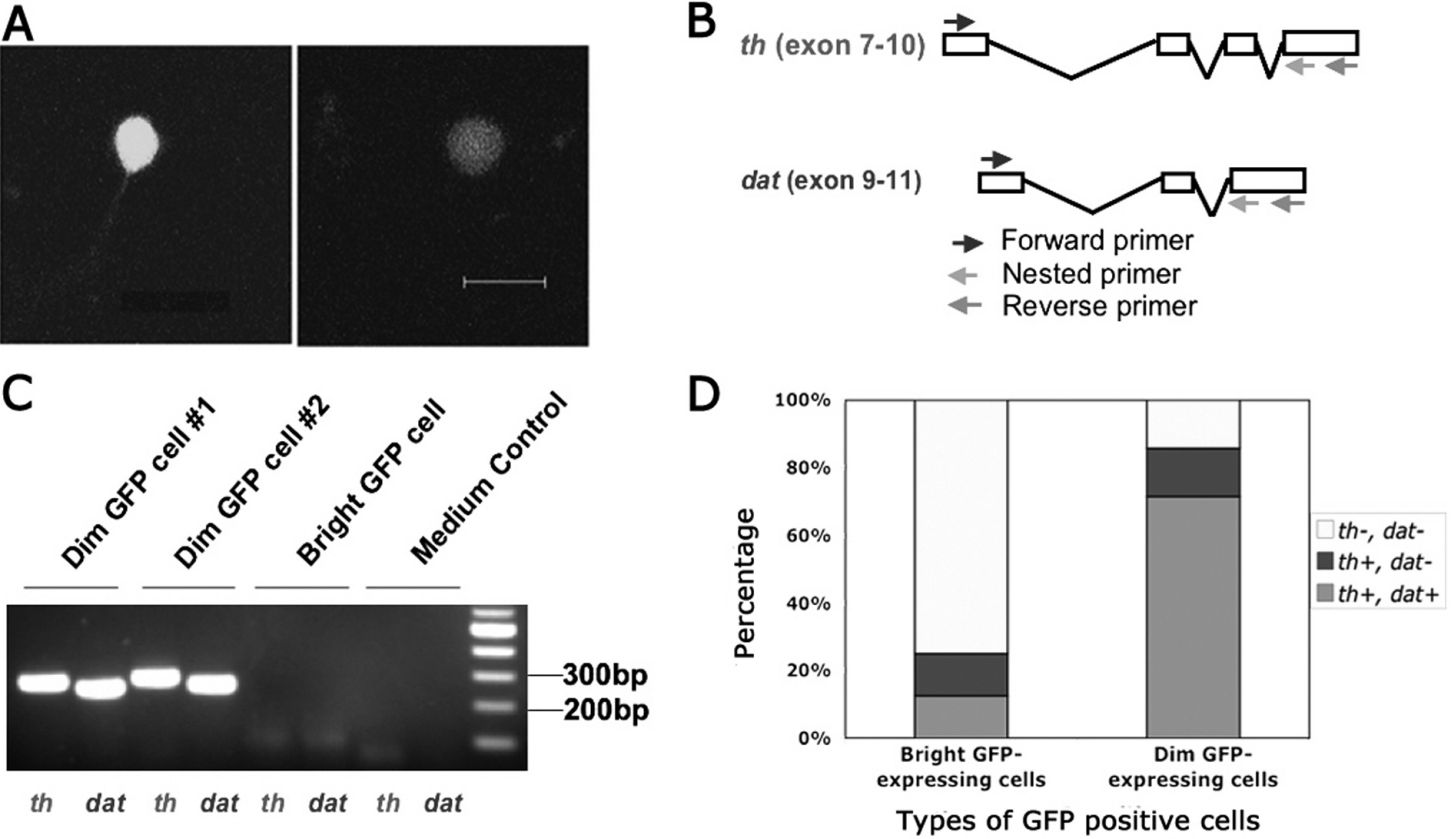
Analysis of two subpopulations of GFP-positive cells using single-cell RT–PCR. **A:** Representative bright and dim GFP-positive cells in dispersed retinal culture were imaged at the same excitation intensity. Scale bar equals 10 μm. **B:** Schematic diagram shows the primers used for seminested PCR. For *th*, the size of the 2nd round PCR product was 277 bp. For *dat*, the size was 250 bp. **C:** Representative gel image shows the 2nd round PCR product from dim GFP cells, bright GFP cells, and medium control sample. Primer sets used were indicated at the bottom. **D:** Expression frequency of *th* and *dat* genes is different between bright GFP expressing cells and dim GFP expressing cells as assayed by single-cell RT–PCR. For bright GFP-expressing cells, 8 cells were examined. For dim GFP-expressing cells, 7 cells were examined.

We noted a marked variation in GFP intensity across labeled neurons and that there was a trend toward a greater incidence of colocalization with TH for dimmer GFP+ cells, whereas the neighboring bright GFP-stained cells were often single labeled. Average gray value analysis using MetaMorph on confocal images of GFP-immunostained whole-mount retinas indicated a twofold difference in fluorescent intensity between bright GFP- and dim GFP-expressing cells (Figure 5). While these two subpopulations were distinguishable in fluorescence intensity (Figure 6A), they exhibited similar cell size and shape.

### Expression of *th* and *dopamine transporter* mRNA in GFP-labeled cells

To further assess what proportion of GFP-expressing cells are dopaminergic, and to test the possibility that molecular components of GFP cells could be analyzed in vitro, we sought to detect *th* and *dat* expression in isolated living cells. We acutely dissociated the retinas and plated retinal cells into culture dishes, and performed single-cell RT–PCR, using primers specific for *th* and *dat*, markers for DA neurons. To increase reaction specificity, seminested PCR primers spanning different exons of the genes were used (Figure 6B). Bright or dim GFP-expressing cells were collected and analyzed by reverse transcription followed by seminested PCR. Amplicons from the second round of PCR were visualized on agarose gels stained with ethidium bromide (Figure 6C). Among the seven dim GFP-expressing cells collected, five cells expressed both *th* and *dat*, while only one out of eight bright GFP-expressing cells did (Figure 6D). These results showed that most dim GFP-expressing cells coexpressed both dopamine-cell marker genes.

### Spontaneous spike activity of GFP-expressing neurons in situ

**Figure 7:**
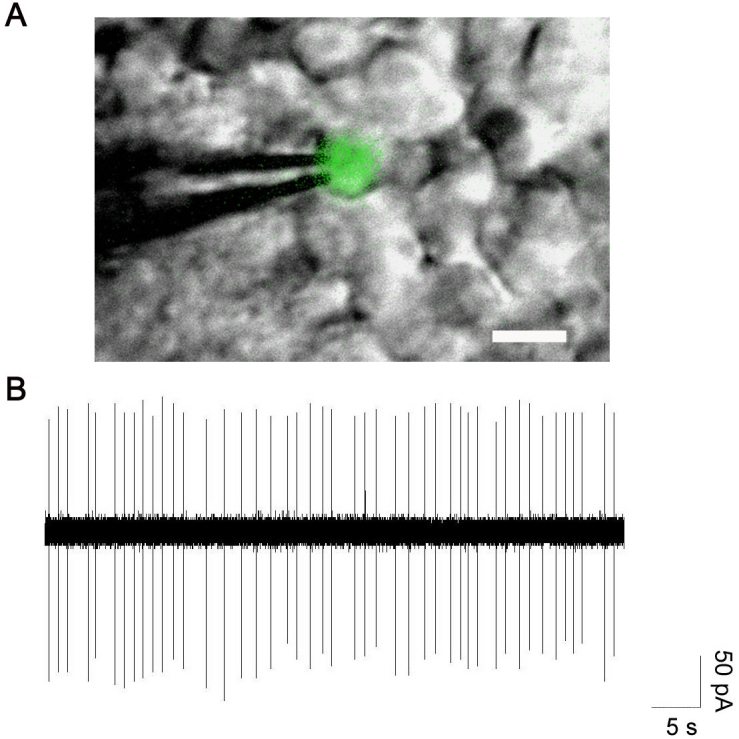
In isolated whole mount retina, GFP-labeled neurons exhibit spontaneous spikes. Merged fluorescence and infrared images of a *Tg(−12^th^:MmGFP)* neuron and recording electrode in a whole mount zebrafish retina is shown in panel **A**. Scale bar equals 10 μm. Panel **B** displays spontaneous spikes recorded in a GFP-labeled cell. Loose-patch recordings were made using a voltage-clamp mode with an electrode holding potential of 0 mV.

Mammalian dopaminergic amacrine cells generate spontaneous action potentials in dissociated culture or in whole mount mouse retinas [15,25,26]. To examine the neuronal activity of dopaminergic cells in the *Tg(−12th:MmGFP)* zebrafish retina, we performed extracellular loose-patch recordings on GFP-expressing cells. Based on the initial observation that most dim-GFP-expressing cells are dopaminergic neurons, for recording we prioritized cells with relatively low GFP expression (see Figure 7A for overlaid fluorescent and DIC images of an example cell). Among the 20 GFP+ cells, 11 exhibited spontaneous spikes. Figure 7B shows a typical recording from a GFP+ cell. The cell fired in single-spike pattern with a spiking rate of 0.75 Hz. For the 11 cells exhibiting spontaneous spikes, the firing rate ranged from 0.47 - 4.70 Hz with an average of 1.48±1.17 Hz (mean ± SD).

## Discussion

We report here the characterization of GFP-expressing retinal neurons in a transgenic zebrafish line where the fluorescent reporter is driven by sequences of the zebrafish *th1* promoter. In this transgenic line, *th1*-driven GFP exhibited robust expression in the brain and retina, but was not restricted to DA neurons in the retina. In juvenile and adult zebrafish retinas, about 30% of GFP-expressing cells were found to be dopaminergic cells by co-labeling with TH. Single-cell RT–PCR results further indicated that most dim GFP-expressing cells were dopaminergic neurons. In addition, spontaneous action potentials were observed in many of labeled cells in darkness, suggesting that these cells are functionally active. These results indicate that this transgenic line, though not a completely specific reporter for retinal dopaminergic neurons, provides the means to enrich for zebrafish retinal dopaminergic neurons in experiments using the living retina and in primary cell culture.

A similar strategy for labeling zebrafish dopaminergic neurons has been reported by Gao et al. [27]. In their transgenic line, GFP expression was driven by the rat *TH* promoter and was present in many more neurons throughout one to two cell layers in the INL, as well as in the ganglion cell layer. In our line, GFP driven by the zebrafish *TH* promoter was restricted to a limited number (approximately 1000/retina) of neurons in the INL only. The zebrafish *TH* promoter used in the present study may more faithfully drive reporter expression compared to the mammalian promoter. It is possible that the presence of different transcriptional regulators in teleost and mammalian organisms is responsible for the disparate transcriptional regulation of these transgenes.

In our *Tg(−12th:MmGFP)* transgenic line, about one-third of the GFP-expressing retinal neurons are TH-IR, while the rest remain to be identified. One possible explanation for this is that the 12 kb zebrafish *TH* promoter may not include all the 5′ regulatory elements, or that there are downstream regulatory elements not included in the transgene, so that GFP is ectopically expressed in some non-DA cells. A silencer or negative regulatory element that would normally suppress TH expression might be missing from the relatively small constructs, as has been proposed to be a potential reason for ectopic transgene expression in the mouse and in the zebrafish [28,29]. Another possibility is that GFP-expressing, TH-negative cells could be non-DA catecholaminergic (CA) cells, referred to as Type 2 CA cells in the mouse and other mammalian species, that have been shown to express TH-promoter driven reporters, but to lack sufficient TH protein to be detected by antibody [15,30]. In mammals, there are distinct differences between DA cells and Type 2 cells in soma size, process morphology, and distribution pattern throughout the retina. However, our results indicate no significant differences in cell size or shape between GFP-TH colocalized cells and GFP single-labeled cells. Instead, we found a fluorescent intensity difference between individual labeled neurons that correlated with DAT expression. Although the identity of the non-DA cells is unknown to us, it is important to note that the GFP-fluorescent intensity can be used to reliably identify DA cells for molecular and physiologic analysis.

Our single-cell RT–PCR results with bright and dim subpopulations of GFP-labeled neurons revealed a useful strategy for identifying dopaminergic neurons for future in vitro and in situ studies. Our results also indicate the possibility of analyzing the molecular components of GFP+ neurons at the single-cell level. For example, DA neurons in the mouse have been reported to express circadian clock genes, as well as multiple subunits of GABA_A_ receptors in the mouse [24,31,32]. Investigation of these genes in the current *Tg(−12th:MmGFP)* fish will provide the information about teleost retinas from a comparative aspect.

Electrophysiology recordings of GFP-expressing cells demonstrated that in the dark, many of these cells generate spontaneous action potentials. Spontaneous activity of dopaminergic retinal neurons has been implicated in the maintenance of the basal level of dopamine release from fish IPCs in a calcium-dependent manner [2]. After prolonged darkness, fish cone horizontal cell responsiveness is suppressed, as is receptive field size of cone horizontal cells, both due to dopamine release [33,34]. These facts suggest that dopamine, released by spontaneous oscillatory spiking of DA-IPCs, modulates the dark-adaptation process of cone horizontal cells. Since horizontal cells mediate lateral inhibitory effects in the OPL and form the antagonistic surround responses of cones, bipolar cells, and certain ganglion cells, dopamine may have a broader effect on dark-adaptation in the retina via cone horizontal cells. In addition, Li and Dowling [35] studied effects of dopamine depletion in zebrafish and found that in DA-cell depleted fish, rod signals were blocked in the inner plexiform layer during dark adaptation. They suggested this rod signaling defect was due to the lack of dopamine release, which is required for the rod signal to be transmitted from the rod to cone bipolar cells in darkness. Our results provide direct evidence of dopaminergic cell activity in darkness in the zebrafish retina. Compared to dopaminergic neurons in the mouse [26], zebrafish dopaminergic neurons exhibit lower rates of spontaneous activity in the dark, similar to the reduced number and frequency of spontaneously active ganglion cells in zebrafish [36]. Although the *TH*:RFP mouse model has been highly successful in defining novel aspects of dopamine amacrine cell control circuitry [26], the zebrafish provides a potentially valuable alternative. Whereas the mouse has a nocturnal, rod-dominated retina, unlike the human, the zebrafish has a diurnal cone-dominated retina. Having molecular and physiologic data on dopaminergic neurons from the zebrafish retina for comparison should allow for elucidation of general principles of retinal dopaminergic organization.

In summary, our results indicate that by using this *Tg(−12th:MmGFP)* transgenic line, we can experimentally enrich for dopaminergic neurons in vitro and in vivo. *Tg(−12th:MmGFP)* targeting of DA retinal neurons can be used as a valuable approach for developmental and functional studies of dopaminergic cells in the zebrafish retina.

## References

[1] Witkovsky P,, Dearry A (1991). Functional roles of dopamine in the vertebrate retina.. Prog Retinal Res.

[2] Djamgoz MB, Wagner HJ (1992). Localization and function of dopamine in the adult vertebrate retina.. Neurochem Int.

[3] Mangel SC (2001). Circadian clock regulation of neuronal light responses in the vertebrate retina.. Prog Brain Res.

[4] Ribelayga C, Wang Y, Mangel SC (2002). Dopamine mediates circadian clock regulation of rod and cone input to fish retinal horizontal cells.. J Physiol.

[5] Sakamoto K, Liu C, Kasamatsu M, Pozdeyev NV, Iuvone PM, Tosini G (2005). Dopamine regulates melanopsin mRNA expression in intrinsically photosensitive retinal ganglion cells.. Eur J Neurosci.

[6] Yamauchi T, Kashiia S, Yasuyoshia H, Zhanga S, Hondaa Y, Ujiharab H, Akaikeb A (2003). Inhibition of glutamate-induced nitric oxide synthase activation by dopamine in cultured rat retinal neurons.. Neurosci Lett.

[7] Linden R, Martins RA, Silveira MS (2005). Control of programmed cell death by neurotransmitters and neuropeptides in the developing mammalian retina.. Prog Retin Eye Res.

[8] Dowling JE, Ehinger B (1975). Synaptic organization of the amine-containing interplexiform cells of the goldfish and Cebus monkey retinas.. Science.

[9] Dowling JE, Ehinger B (1978). The interplexiform cell system. I. Synapses of the dopaminergic neurons of the goldfish retina.. Proc R Soc Lond B Biol Sci.

[10] Dowling JE (1991). Retinal neuromodulation: the role of dopamine.. Vis Neurosci.

[11] Zucker CL, Dowling JE (1987). Centrifugal fibres synapse on dopaminergic interplexiform cells in the teleost retina.. Nature.

[12] Huang L, Maaswinkel H, Li L (2005). Olfactoretinal centrifugal input modulates zebrafish retinal ganglion cell activity: a possible role for dopamine-mediated Ca2+ signalling pathways.. J Physiol.

[13] Negishi K, Kato S, Teranishi T (1981). Indoleamine-accumulating cells and dopaminergic cells are distributed similarly in carp retina.. Neurosci Lett.

[14] Versaux-Botteri C, Nguyen-Legros J, Vigny A, Raoux N (1984). Morphology, density and distribution of tyrosine hydroxylase-like immunoreactive cells in the retina of mice.. Brain Res.

[15] Gustincich S, Feigenspan A, Wu DK, Koopman LJ, Raviola E (1997). Control of dopamine release in the retina: a transgenic approach to neural networks.. Neuron.

[16] Zhang DQ, Stone JF, Zhou T, Ohta H, McMahon DG (2004). Characterization of genetically labeled catecholamine neurons in the mouse retina.. Neuroreport.

[17] Holzschuh J, Ryu S, Aberger F, Driever W (2001). Dopamine transporter expression distinguishes dopaminergic neurons from other catecholaminergic neurons in the developing zebrafish embryo.. Mech Dev.

[18] Arenzana FJ, Arévalo R, Sánchez-González R, Clemente D, Aijón J, Porteros A (2006). Tyrosine hydroxylase immunoreactivity in the developing visual pathway of the zebrafish.. Anat Embryol (Berl).

[19] Guo S, Wilson SW, Cooke S, Chitnis AB, Driever W, Rosenthal A (1999). Mutations in the zebrafish unmask shared regulatory pathways controlling the development of catecholaminergic neurons.. Dev Biol.

[20] Guo S, Yamaguchi Y, Schilbach S, Wada T, Lee J, Goddard A, French D, Handa H, Rosenthal A (2000). A regulator of transcriptional elongation controls vertebrate neuronal development.. Nature.

[21] Holzschuh J, Barrallo-Gimeno A, Ettl AK, Durr K, Knapik EW, Driever W (2003). Noradrenergic neurons in the zebrafish hindbrain are induced by retinoic acid and require tfap2a for expression of the neurotransmitter phenotype.. Development.

[22] Ettl AK, Holzschuh J, Driever W (2006). The zebrafish mutation m865 affects formation of dopaminergic neurons and neuronal survival, and maps to a genetic interval containing the sepiapterin reductase locus.. Anat Embryol (Berl).

[23] Ryu S, Mahler J, Acampora D, Holzschuh J, Erhardt S, Omodei D, Simeone A, Driever W (2007). Orthopedia homeodomain protein is essential for diencephalic dopaminergic neuron development.. Curr Biol.

[24] Ruan GX, Zhang DQ, Zhou T, Yamazaki S, McMahon DG (2006). Circadian organization of the mammalian retina.. Proc Natl Acad Sci USA.

[25] Feigenspan A, Gustincich S, Bean BP, Raviola E (1998). Spontaneous activity of solitary dopaminergic cells of the retina.. J Neurosci.

[26] Zhang DQ, Zhou TR, McMahon DG (2007). Functional heterogeneity of retinal dopaminergic neurons underlying their multiple roles in vision.. J Neurosci.

[27] Gao Y, Li P, Li L (2005). Transgenic zebrafish that express tyrosine hydroxylase promoter in inner retinal cells.. Dev Dyn.

[28] Matsushita N, Okada H, Yasoshima Y, Takahashi K, Kiuchi K, Kobayashi K (2002). Dynamics of tyrosine hydroxylase promoter activity during midbrain dopaminergic neuron development.. J Neurochem.

[29] Jessen JR, Meng A, McFarlane RJ, Paw BH, Zon LI, Smith GR, Lin S (1998). Modification of bacterial artificial chromosomes through chi-stimulated homologous recombination and its application in zebrafish transgenesis.. Proc Natl Acad Sci USA.

[30] Versaux-Botteri C, Martin-Martinelli E, Nguyen-Legros J, Geffard M, Vigny A, Denoroy L (1986). Regional specialization of the rat retina: catecholamine-containing amacrine cell characterization and distribution.. J Comp Neurol.

[31] Gustincich S, Contini M, Gariboldi M, Puopolo M, Kadota K, Bono H, LeMieux J, Walsh P, Carninci P, Hayashizaki Y, Okazaki Y, Raviola E (2004). Gene discovery in genetically labeled single dopaminergic neurons of the retina.. Proc Natl Acad Sci USA.

[32] Gustincich S, Feigenspan A, Sieghart W, Raviola E (1999). Composition of the GABA(A) receptors of retinal dopaminergic neurons.. J Neurosci.

[33] Tornqvist K, Yang XL, Dowling JE (1988). Modulation of cone horizontal cell activity in the teleost fish retina. III. Effects of prolonged darkness and dopamine on electrical coupling between horizontal cells.. J Neurosci.

[34] Mangel SC, Dowling JE (1987). The interplexiform-horizontal cell system of the fish retina: effects of dopamine, light stimulation and time in the dark.. Proc R Soc Lond B Biol Sci.

[35] Li L, Dowling JE (2000). Disruption of the olfactoretinal centrifugal pathway may relate to the visual system defect in night blindness b mutant zebrafish.. J Neurosci.

[36] Emran F, Rihel J, Adolph AR, Wong KY, Kraves S (2007). Dowling JE. OFF ganglion cells cannot drive the optokinetic reflex in zebrafish.. Proc Natl Acad Sci USA.

